# Antimicrobial susceptibilities of *Neisseria gonorrhoeae* in Canada, 2021

**DOI:** 10.14745/ccdr.v49i09a05

**Published:** 2023-09-01

**Authors:** Pamela Sawatzky, Brigitte Lefebvre, Mathew Diggle, Linda Hoang, Jason Wong, Samir Patel, Paul Van Caessele, Jessica Minion, Richard Garceau, Sarah Jeffrey, David Haldane, Lillian Lourenco, Genevieve Gravel, Michael Mulvey, Irene Martin

**Affiliations:** 1 National Microbiology Laboratory Branch, Public Health Agency of Canada, Winnipeg, MB; 2 Laboratoire de santé publique du Québec, Ste-Anne-de- Bellevue, QC; 3 Provincial Laboratory for Public Health, Edmonton, AB; 4 BC Centre for Disease Control Public Health Laboratory, Vancouver, BC; 5 Public Health Ontario Laboratory, Toronto, ON; 6 Cadham Provincial Laboratory, Winnipeg, MB; 7 Roy Romanow Provincial Laboratory, Regina, SK; 8 Dr. Georges-L.-Dumont University Hospital Centre, Moncton, NB; 9 Government of Northwest Territories, Yellowknife, NT; 10 Queen Elizabeth II Health Sciences Centre, Halifax, NS; 11 Centre for Communicable Diseases and Infection Control Branch, Public Health Agency of Canada, Ottawa, ON

**Keywords:** gonorrhea, *Neisseria gonorrhoeae*, antimicrobial resistance, antimicrobial susceptibility, national surveillance system, passive surveillance

## Abstract

**Background:**

In Canada, gonorrhea is the second most prevalent bacterial sexually transmitted infection. The Gonococcal Antimicrobial Surveillance Programme (GASP - Canada), a passive surveillance system monitoring antimicrobial resistance in *Neisseria gonorrhoeae* in Canada since 1985, is the source for this summary of demographics, antimicrobial resistance and *N. gonorrhoeae* multi-antigen sequence typing (NG-MAST) of gonococcal isolates collected in Canada in 2021.

**Methods:**

Provincial and territorial public health laboratories submitted *N. gonorrhoeae* cultures and data to the National Microbiology Laboratory in Winnipeg as part of the surveillance system. The antimicrobial resistance and molecular type of each isolate received were determined.

**Results:**

In total, 3,439 *N. gonorrhoeae* cultures were received from laboratories across Canada in 2021, a 9.9% increase since 2020 (n=3,130). Decreased susceptibility to cefixime increased significantly (*p*<0.001) in 2021 (1.5%) compared to 2017 (0.6%). No significant change in decreased susceptibility to ceftriaxone was detected between 2017 and 2021 (0.6%) (*p*>0.001); however, one ceftriaxone-resistant isolate was identified. Azithromycin resistance decreased significantly (*p*<0.001) in 2021 (7.6%) compared to 2017 (11.7%); however, there was a significant increase (*p*<0.001) in the proportion of cultures with an azithromycin minimum inhibitory concentration of at least 1 mg/L (2017=22.2% to 2021=28.1%). In 2021, NG-MAST-19875 (15.3%) was the most prevalent sequence type in Canada; 20.3% of isolates with this sequence type were resistant to azithromycin.

**Conclusion:**

The spread of antimicrobial-resistant gonorrhea is a significant public health concern. The continued regional and national surveillance of antimicrobial resistance in *N. gonorrhoeae* is essential in ensuring effective treatment therapies are recommended.

## Introduction

Gonorrhea, caused by *Neisseria gonorrhoeae,* is the second most reported bacterial sexually transmitted infection (STI) in Canada. It causes urethritis in males and while in females it is often asymptomatic, it can present as cervicitis and lead to serious complications such as infertility and pelvic inflammatory disease ((1)). Left untreated, disseminated gonococcal infections (DGI) may occur if the bacterium enters the blood and other sterile sites. Disseminated gonococcal infections have not been considered common in Canada but have increased from 2017 to 2021 ((2)) and can cause arthritis, dermatitis, migratory polyarthralgia, tenosynovitis and, in rare cases, endocarditis ((3,4)).

Canada reported 30,883 cases of gonorrhea in 2020 ((2,5)). This is slightly lower than the number reported in 2019 (n=35,443), likely due to the effects of coronavirus disease 2019 (COVID-19) on public health care ((2,5,6)). Even with the decrease in reported gonorrhea cases, the 2020 gonorrhea rate (80.1 per 100,000 population) is twice the rate reported in 2013 (40.56 per 100,000 population) ((1)).

*Neisseria gonorrhoeae* has constantly evolved to resist antimicrobials used for gonorrhea treatment. The World Health Organization’s (WHO) Global Action Plan was released with the objective to control the spread and minimize the impact of antimicrobial resistant *N. gonorrhoeae* ((3,4)). The Public Health Agency of Canada currently recommended treatment regimen of ceftriaxone 250 mg intramuscularly plus azithromycin 1 g orally ((7)) is at risk due to persistent azithromycin resistance (AziR) and the decreased susceptibility (DS) to cephalosporins observed among Canadian *N. gonorrhoeae* isolates. Cases of cephalosporin-resistant *N. gonorrhoeae* were identified in Canada between 2017 and 2021 ((2,8,9)) and AziR has increased beyond the 5% resistance cut-off recommended by the WHO to trigger a review of current recommended therapies ((4)).

The Gonococcal Antimicrobial Surveillance Program (GASP - Canada) is a passive national surveillance program that has been operating since 1985. Antimicrobial susceptibility testing (AST) and molecular characterization using *N. gonorrhoeae* multi-antigen sequence typing (NG-MAST) are performed on isolates that are submitted to GASP - Canada. The NG-MAST is highly distinctive and can be used to investigate treatment failures and outbreaks and NG-MAST sequence types (ST) have also shown a close association with antimicrobial resistance (AMR) ((10–12)).

Gonorrhea is an important public health concern with its ability to cause infertility, pelvic inflammatory disease and DGI ((13,14)). The capacity for *N. gonorrhoeae* to constantly evolve to resist antimicrobials means that continued surveillance is necessary to ensure treatment therapies are effective against currently circulating strains and to slow the spread of AMR strains.

As in 2020, decreased testing capacity of laboratories across Canada for *N. gonorrhoeae* cultures due to the severe acute respiratory syndrome coronavirus 2 (SARS-CoV-2) pandemic contributed towards a considerable decrease in the number of isolates received by GASP - Canada in 2021 and included in this report compared to previous years. This report summarizes the AMR trends and molecular types of *N. gonorrhoeae* cultures in Canada from 2017 to 2021.

## Methods

### Surveillance

As part of GASP - Canada, provincial and territorial partners voluntarily send *N. gonorrhoeae* cultures to the National Microbiology Laboratory (NML) predominantly when the provincial laboratories detect resistance/decreased susceptibility to at least one antimicrobial or if the provincial laboratories do not perform AST. Commencing in 2019, certain provinces’ AST data (in the form of minimum inhibitory concentrations, MICs) of isolates not sent to NML for testing were used in our analysis in conjunction with MICs of isolates tested at NML. Alberta sends all their resistant gonorrhea cultures (n=652 in 2021) to NML for testing and submits their AST data for the remaining isolates (n=131 in 2021). Québec (n=985 in 2021) and British Columbia (n=119 in 2021) send isolates to NML that adhere to the following criteria: 1) resistant to azithromycin; 2) decreased susceptibility to cefixime and/or ceftriaxone; 3) approaching resistance/decreased susceptibility to these antimicrobials. These provinces submit AST and patient data for the remaining isolates tested: Québec (n=576) and British Columbia (n=210 in 2021). Ontario sends to NML all resistant isolates (n=250 in 2021) and informs NML of the total number tested in their province (n=636 in 2021). Manitoba (n=44 in 2021), New Brunswick (n=32 in 2021) and Saskatchewan (n=41 in 2021) send all isolates cultured if possible. Nova Scotia, Newfoundland and Labrador and the Northwest Territories also send all their gonorrhea cultures to NML (n=13 in 2021). A total of 3,439 *N. gonorrhoeae* isolates were cultured across Canada in 2021: 2,006 unique, viable cultures were submitted to NML for AST and molecular typing. The AST results as determined by provincial and territorial laboratories and patient demographics for another 903 cultures were submitted to NML. The remaining 530 cultures were tested by provincial and territorial laboratories and were recorded as susceptible by NML, as no AST or demographic data were submitted. **Table S1** includes the number of cultures submitted from each province or territory and the number of cultures with resistance to at least one antimicrobial. The total number of *N. gonorrhoeae* isolates tested across Canada was 3,439 and this was used as the denominator in resistance calculations, unless otherwise noted.

### Isolate testing

Antimicrobial susceptibility testing using agar dilution ((15)) and/or whole genome sequencing (WGS) methods ((11)) was performed on all *N. gonorrhoeae* cultures received by NML (n=2,006). Minimum inhibitory concentrations for 10 antimicrobials were determined and interpretation of results were based on the Clinical and Laboratory Standards Institute for five of them (penicillin, tetracycline and azithromycin all resistant (R) when MIC is at least 2 mg/L; ciprofloxacin R when MIC is at least 1 mg/L; spectinomycin R when MIC is at least 128 mg/L) ((14)). The WHO guidelines were used for ceftriaxone (DS when MIC is at least 0.125 mg/L) and cefixime (DS when MIC is at least 0.25 mg/L) ((4)). Erythromycin (R when MIC is at least 2 mg/L), ertapenem (non-susceptible when MIC is at least 0.063 mg/L) and gentamicin (R when MIC is at least 32 mg/L) interpretations are based on publications ((16–19)) (**Table S2**). Testing of ß-lactamase was performed on all cultures. Cultures with tetracycline MICs of at least 16 mg/L were tested for the *tetM* plasmid by polymerase chain reaction ((20)). Isolates were classified as susceptible, resistant, multi-drug resistant gonoccoci (MDR-GC, either DS or R to one recommended gonorrhea therapy at the time of the analysis, plus resistance to at least two other antimicrobials) or extensively drug-resistant gonococci (XDR-GC, either DS or R to two recommended gonorrhea therapies at the time of analysis, plus resistance to at least two other antimicrobials).

Genotyping of cultures was determined by NG-MAST using polymerase chain reaction ((12)) and/or WGS ((11)). SeqMan Pro 15 (DNAStar, Madison, Wisconsin) was used to assemble strands of Sanger-sequenced DNA and the ST was determined when sequences were submitted to the PubMLST *Neisseria* spp. database. The previous NG-MAST website (http://www.ng-mast.net) was decommissioned and several thousand previously identified STs were deleted. Therefore, some allelic profiles from previous years were updated with new STs in this report.

### Whole genome sequencing

The DNA from isolates on which WGS was successfully performed (n=1,231) was prepared using the Epicentre Masterpure Complete DNA and RNA Extraction Kit (Mandel Scientific, Guelph, Ontario). Briefly, the sequencing method used involved creating libraries (using Nextera sample preparation kits [Illumina, San Diego, California]) with 300 bp paired-end index reads generated on the Illumina NextSeq platform (Illumina). Galaxy Version 1.0.4+galaxy was used to assess the quality of the reads, assemble them and analyze single nucleotide variants with NCCP1145 (GenBank accession number NC_011035) as the mapping reference. Whole genome sequencing data was used to detect molecular AMR markers and to determine multi-locus sequence type (MLST), *Neisseria gonorrhoeae* sequence typing for antimicrobial resistance (NG-STAR) and NG-MAST STs ((11)).

### Data analysis

Age, sex, isolation site, province and date of collection were provided with the *N. gonorrhoeae* isolates. Duplicate isolates were identified and removed from the denominator if multiple isolates from the same patient had the same ST and were collected within four weeks of each other. A hierarchy of isolation sites was used to determine which isolates were considered duplicates, the order of which was 1) sterile site (DGI), 2) throat, 3) rectal and 4) urogenital. Each figure includes the denominator used in its description. Trends of AMR and STs were determined nationally. Azithromycin resistance and cefixime and ceftriaxone DS (CeDS and CxDS, respectively) were also analyzed at the provincial or territorial level. Correlation of the most common STs with AMR was also indicated. Comparisons of AMR proportions were made using the Fisher’s exact test with a 99% confidence interval employing EpiCalc 2000 (version 1.02; Brixton Health).

## Results

### Isolates tested, demographics and isolation sites

**Figure 1:**
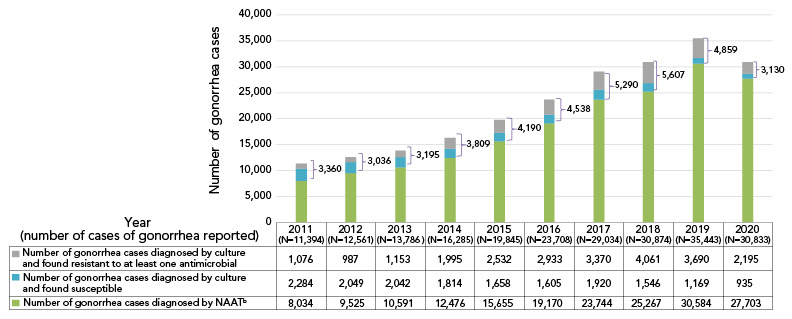
Reported *Neisseria gonorrhoeae* cases in Canada, 2011–2020^a,b^ Abbreviation: NAAT, nucleic acid amplification testing ^a^ Approximately 10% of all gonorrhea cases were diagnosed by culture in Canada in 2020. The rest was detected using nucleic acid amplification test technology. The number of reported cases for 2021 had not yet been determined at the time of publication ^b^ The number of gonorrhea cases diagnosed by nucleic acid amplification testing is determined by subtracting the number of cultures tested across Canada from the number of gonorrhea cases reported ((1))

In 2021, 3,439 *N. gonorrhoeae* isolates were tested across Canada. Over 70% (72.7%, n=2,501/3,349) were resistant to at least one antibiotic (Table S1). This proportion does not include the gonorrhoeae cases that were diagnosed using nucleic acid amplification tests (NAATs). Cases diagnosed by NAATs are not routinely tested for AMR and accounted for 90% of diagnosed and reported gonorrhea cases in Canada in 2020 (**Figure 1**).

Age, sex and isolation site information were submitted to NML for 2,909 cultures in 2021. Over 70% (71.2%, n=2,072/2,909) of *N. gonorrhoeae* cultures were from individuals between the ages of 21 and 40 years; 21.3% (n=620/2,909) from persons 41 years and older; 7.4% (n=214/2,909) from those younger than 21 years. Isolates were primarily from males 84.1% (n=2,446/2,909), with 15.0% (n=436/2,909) from females and 0.9% (n=27/2,909) from patients who are either gender-diverse or whose gender was not given. The prevalent isolation site in males was the penis/urethra (57.0%, n=1,395/2,446) and for females it was the throat (33.9%, n=148/436). See **Table S3** for more details.

### Cephalosporin antimicrobial trends in Canada, 2017–2021

**Figure 2:**
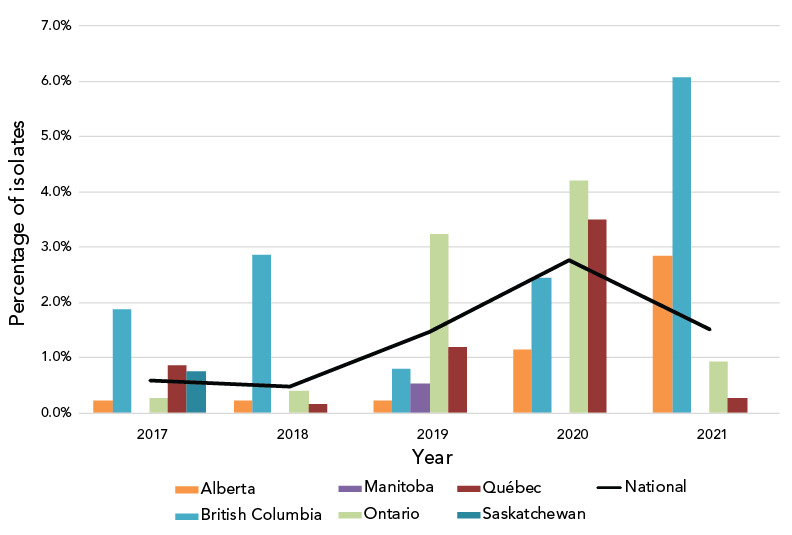
Percentage of *Neisseria gonorrhoeae* cultures with decreased susceptibility to cefixime by province, 2017–2021^a,b^ ^a^ Provinces included in this figure are only those that submitted at least one culture to the National Microbiology Laboratory that had decreased susceptibility to cefixime ^b^ Denominators used for the calculations of the percentages are the number of cultures tested in each province (**Table S4**)

There is a significant increase in CeDS (MIC of at least 0.25 mg/L), from 0.6% in 2017 to 1.5% in 2021 (*p*<0.001), and a significant decrease from the 2.8% reported in 2020 (*p*<0.001) (**Figure 2**).

**Figure 3:**
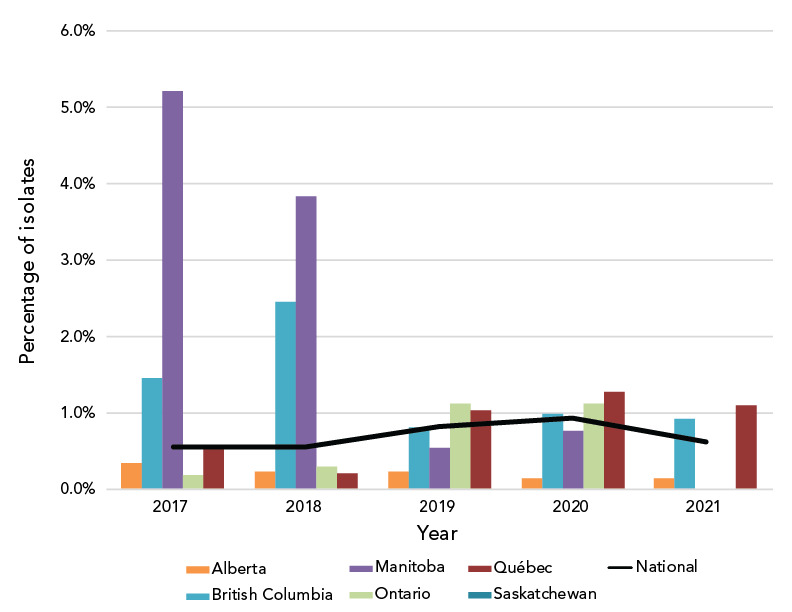
Percentage of *Neisseria gonorrhoeae* cultures with decreased susceptibility to ceftriaxone by province, 2017–2021^a,b^ ^a^ Provinces included in this figure are only those that submitted at least one culture to the National Microbiology Laboratory that had decreased susceptibility to ceftriaxone ^b^ Denominators used for the calculations of the percentages are the number of cultures tested in each province (Table S4)

Decreased susceptibility to ceftriaxone (CxDS, MIC of at least 0.125 mg/L) has not seen a significant change since 2017, ranging from 0.55% in 2017 and 2018 to 0.93% in 2020 and declining to 0.61% (n=21/3,349) in 2021 (**Figure 3**). Of note, one isolate was ceftriaxone-resistant (CxR) with an MIC of 1 mg/L, while the remaining 20 isolates classified as CxDS had MICs of 0.125 mg/L. The CxR isolate was isolated in British Columbia in October 2021 from a 25-year-old female. Original treatment with 800 mg cefixime orally failed; however, this was resolved with one intramuscular injection of 250 mg ceftriaxone. The isolate was identified as NG-MAST-19937, MLST-7365 and NG-STAR-3903 with the *penA* allele 60.001 (**Table 1**).

**Table 1:** Ceftriaxone-resistant *Neisseria gonorrhoeae* isolate, 2021

NML #	Province	Collection date	Gender	Age (years)	Isolation site	NG-MAST	Resistance profile	MIC (mg/L)		MLST	*penA*
CX	CE
61829	British Columbia	2021-10-20	Female	25	Vagina	ST-19937	CeDS; CxDS; CipR; EryR; PenR; TetR	1	2	7365	60.001

Abbreviations: CE, cefixime; CeDS, cefixime decreased susceptibility; CipR, ciprofloxacin-resistant; CX, ceftriaxone; CxDS, ceftriaxone decreased susceptibility; EryR, erythromycin-resistant; MIC, minimum inhibitory concentration; MLST, multi-locus sequence type; NG-MAST, *Neisseria gonorrhoeae* multi-antigen sequence type; NML, National Microbiology Laboratory; PenR, penicillin-resistant; ST, sequence type; TetR, tetracycline-resistant

### Azithromycin resistance in Canada, 2017–2021

**Figure 4:**
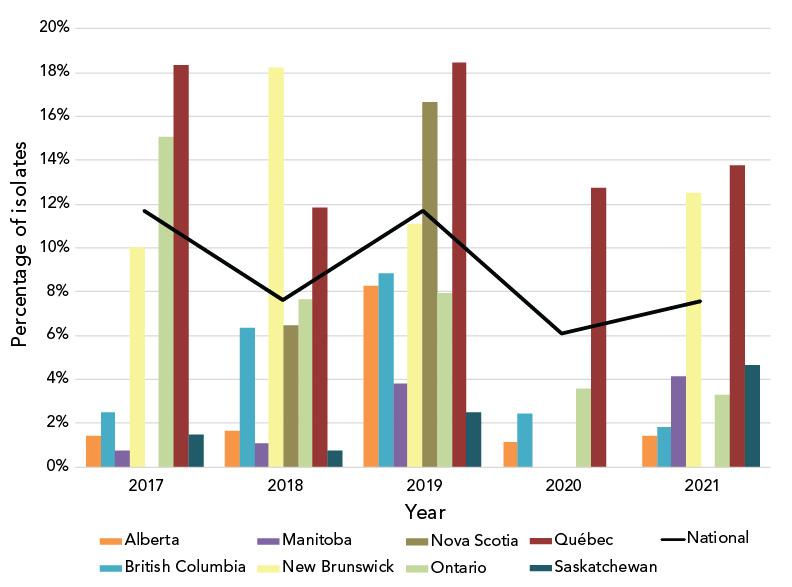
Percentage of azithromycin-resistant *Neisseria gonorrhoeae* cultures by province, 2017-2021^a,b^ ^a^ Provinces included in this figure are only those that submitted at least one culture to the National Microbiology Laboratory that was azithromycin-resistant. Newfoundland and Labrador had one azithromycin-resistant isolate in 2019 ^b^ Denominators used for the calculations of the percentages are the number of cultures tested in each province (Table S4)

**Figure 5:**
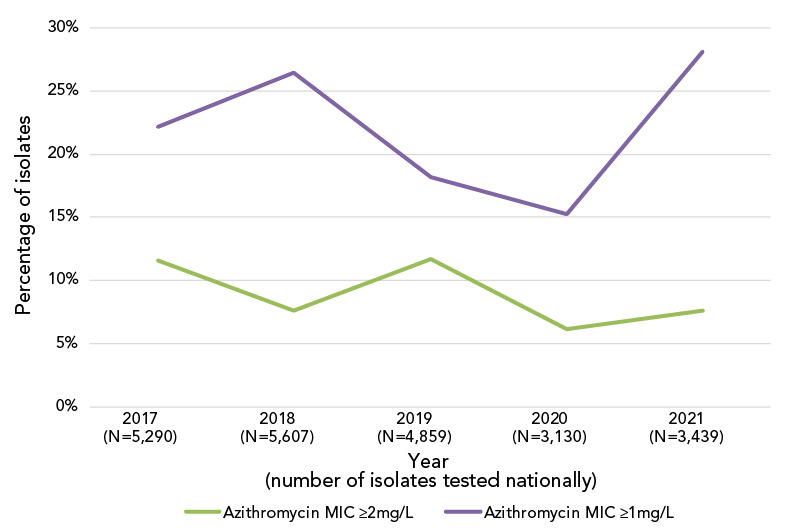
Trends of the percentage of azithromycin minimum inhibitory concentrations for *Neisseria gonorrhoeae* at the susceptibility of breakpoints^a^ ^a^ Breakpoints of at least 1 mg/L and at least 2 mg/L

Azithromycin resistance decreased significantly (*p*<0.001) from 11.7% in 2017 to 7.6% in 2021 (**Figure 4**); however, when a comparison of the proportion of isolates with a MIC of at least 1 mg/L is made between 2017 and 2021, there was a significant increase (*p*<0.001), from 22.2% (n=1,172/5,290) to 28.1% (n=968/3,439) (**Figure 5**).

### Resistance trends in other antimicrobials, 2017–2021

**Figure 6:**
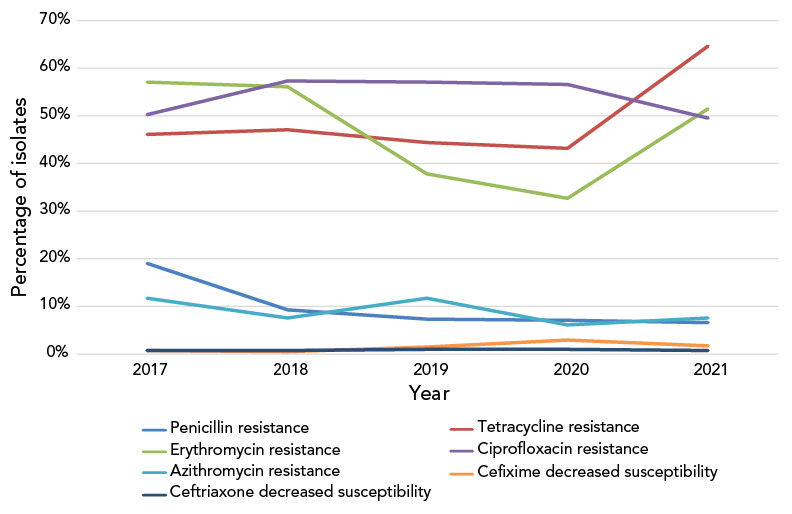
Percentage of antimicrobial resistance of *Neisseria gonorrhoeae* isolates tested in Canada, 2017–2021^a,b^ ^a^ Percentages are based on the total number of isolates tested nationally: 2017=5,290; 2018=5,607; 2019=4,859; 2020=3,130; 2021=3,439 ^b^ Due to some provinces not testing all seven antimicrobials from 2017 to 2021, penicillin denominators were 3,267, 3,883, 3,822, 2,409 and 2,334, respectively; erythromycin denominators were 2,879, 3,418, 3,446, 2,025 and 2,006, respectively. In 2020 and 2021, tetracycline denominators were 2,409 and 2,334, respectively

The proportion of *N. gonorrhoeae* isolates resistant to ciprofloxacin has remained high but stable (between 49% and 57%) from 2017 to 2021. In 2021, tetracycline resistance was at an all-time high of 65.9%, erythromycin resistance was at 51.5% and penicillin resistance was below 7% (**Figure 6**). Non-susceptibility to ertapenem decreased significantly (*p*<0.001) but remained high from 87.2% in 2017 to 62.0% in 2021. Gentamicin resistance has remained at 0%.

### Multi-drug resistant and extensively drug-resistant gonococci in Canada, 2017–2021

The number of MDR cultures decreased significantly (*p*<0.001) between 2017 (12.2%) and 2021 (7.8%) (**Figure S1**). No XDR cultures were identified in Canada in 2021; however, there were 29 XDR-GC isolates identified between 2012 and 2020 (**Figure S2**, **Table S5**).

### Disseminated gonococcal infections cases in Canada, 2017–2021

Between 2016 and 2020, there was a significant increase (*p*<0.001) in both the number and proportion of DGI cases from 0.03% (n=6/23,708) to 0.20% (n=71/30,833) within Canada. In 2021, this proportion decreased slightly (*p*=0.001) compared to 2020, to 0.13% (n=40/30,833). The sources of the DGI in 2021 included synovial fluid (50.0%, n=20/40), blood (45.0%, n=18/40) and eyes, specifically designated as DGI (5.0%, n=2/40). Nine of the DGI (22.5%) were susceptible to all tested antimicrobials, two were resistant to azithromycin and the remaining 29 were resistant to other antimicrobials including erythromycin, tetracycline and ciprofloxacin. None had DS to cephalosporins. Note that the number of cases reported in Canada in 2020 was used as the denominator (n=30,833) to estimate the proportion of DGI amongst cases in 2021.

### *Neisseria gonorrhoeae* multi-antigen sequence typing trends in Canada, 2017–2021

**Figure 7:**
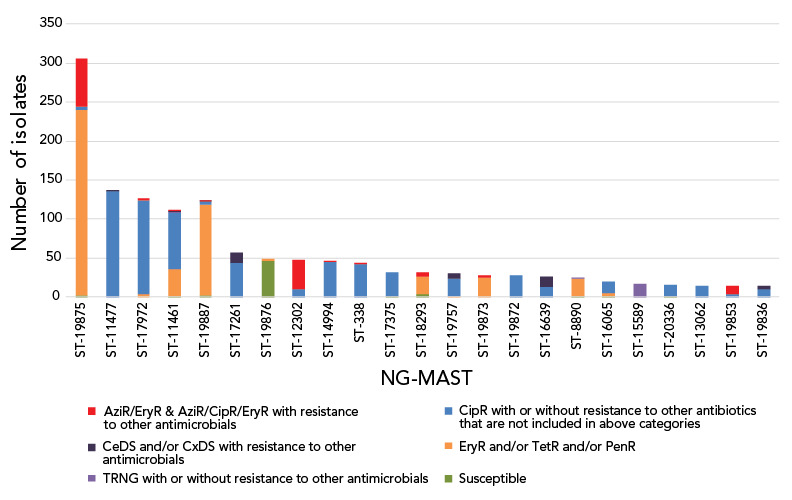
Distribution of resistance characterizations within *Neisseria gonorrhoeae*-multi-antigen sequence typing sequence types, 2021, n=2,006^a^ Abbreviations: AziR, azithromycin-resistant; CeDS, cefixime decreased susceptibility; CipR, ciprofloxacin-resistant; CxDS, ceftriaxone decreased susceptibility; EryR, erythromycin resistant; PenR, penicillin-resistant; ST, sequence type; TetR, tetracycline-resistant; TRNG, tetracycline-resistant *Neisseria gonorrhoeae* ^a^ Figure does not include 33 isolates that were non-typeable. This graph represents 1,338 isolates. The remaining 635 isolates are dispersed among 260 sequence types containing 1–12 isolates each

In 2021, 1,973 out of the 2,006 cultures submitted were successfully typed for NG-MAST. The most frequently detected NG-MAST sequence type in Canada was ST-19875 (n=306), followed by ST-11477 (n=137) and ST-17972 (n=127). Approximately 20% of ST-19875 isolates were identified with AziR, while ST-11477 and ST-17972 isolates were primarily resistant to ciprofloxacin and tetracycline, or ciprofloxacin and erythromycin, respectively (**Figure 7**). **Figure S3** displays the trending of prevalent STs over the last five years. From 2017 to 2020, ST-12302 and ST-14994 were the most prevalent, while in 2021, they were the eighth and ninth most prevalent STs, respectively. While the number of isolates with ST-12302 (n=47) has been decreasing, in 2021, 15 other STs (ST-8890, n=24; ST-19853, n=14; ST-19772, n=11; ST-17629, n=10; ST-19935, n=9; ST-19854, n=9; ST-19866, n=8; ST-20691, n=3; ST-19852, n=3; and ST-14076, ST-20388, ST-19900, ST-19924, ST-8241, ST-20379 all with n=1 each) were identified with two or fewer base pair differences compared to ST-12302. The number of isolates found in this cluster of STs, including ST-12302, was 144; 61.8% (n=89/144) were AziR accounting for 34.1% (n=89/261) of AziR isolated in 2021. The ST-19875 was first identified in 2020 in low numbers (n=22) and only in Québec. In 2021, this ST type has spread to five more provinces (**Figure S4**).

## Discussion

The SARS-CoV-2 global pandemic, declared in 2020 ((6,21)), was still affecting public health care in 2021 ((22)). Reported cases of gonorrhea for 2021 had not been released by the date of publication of this study, but this number dropped from 35,475 cases in 2019 to 30,833 cases in 2020. Reduced testing because of NAAT kit supply shortages ((23)), stay-at-home mandates by public health authorities and hesitancy of infected to seek care contributed to this decrease ((24)). While the number of gonorrhea cultured across Canada increased slightly between 2020 (n=3,130) and 2021 (n=3,439), it is still 30% less than what was seen in 2019 (n=4,859) (Table S1). It is unlikely that this is due to a decrease in infections and more probable that it is due to a decrease in testing ((6,22)). Continued interruptions in testing can cause major increases in the incidence of STIs, including *N. gonorrhoeae,* which may take years to return to levels seen before COVID-19 ((25)). In Canada, a report summarizing the impact of the pandemic on health care stated that since its beginning, people have been more hesitant to seek care ((23)). The negative impacts that the pandemic has had on the healthcare system will take years to correct ((22)). Long-term adverse consequences, such as an increase in pelvic inflammatory disease, DGI and infertility may be the result.

The proportion of isolates in 2021 demonstrating CeDS decreased compared to 2020, although it was higher than in 2017 and 2018. The 2020 higher proportion of isolates with CeDS was primarily caused by isolates identified as ST-16639 in Ontario and Québec. The proportion of this ST decreased from 3.3% (n=53/1,590) in 2020 to 1.3% (n=26/2,006) in 2021.

The increase in CeDS since 2018 could be a result of a potential increase in oral therapy using cefixime (combination therapy of 800 mg cefixime plus 1 g azithromycin) as opposed to the intramuscular injection of ceftriaxone (250 mg ceftriaxone intramuscularly plus 1 g azithromycin orally). Oral therapy does not require a visit to a doctor or clinic during times of limited health services and telehealth appointments. In 2021, fewer restrictions were in place in Canada, allowing in-office visits to resume which may have induced the decrease of CeDS in 2021.

The CxR isolate (isolate ID 61829) with the ceftriaxone MIC of 1 mg/L and the cefixime MIC of 2 mg/L is of concern. It was also resistant to penicillin, tetracycline, erythromycin and ciprofloxacin. Initial treatment of 800 mg orally failed and was followed up with one 250 mg ceftriaxone intramuscular injection. Test of cure confirmed treatment success.

Ceftriaxone-resistant gonococcal isolates have been previously reported in Canada ((8,9)) and globally, including in Japan ((26)), Australia ((27)), China ((28–31)), Denmark ((32)) and Ireland ((33)). Three of the five Canadian CxR isolates reported since 2017, including 61829, have the *penA* allele 60.001 as well as the same AMR-associated mutations (**Table S6**) seen in Japan and Australia (FC428 clone) ((28)). Isolate 61829 has an MLST (7365) that has been seen in a CxR/AziR isolate in China ((31)) but the NG-MAST and NG-STAR types are unique. The United Kingdom reported an isolate with both CxR and high-level AziR that failed treatment in 2018. This isolate had the same AMR-associated mutations as well, with an additional four copies of the A2059G mutation on the 23S rRNA ((34)).

The national levels of AziR in Canada have been inconsistent between 2017 and 2021, shifting between 12% and 6% in alternate years (Figure 4). In response to high levels of AziR, some regions/jurisdictions have updated their recommended treatment to either 250 mg or 500 mg of ceftriaxone intramuscular without azithromycin ((35,36)). Continued surveillance will determine the effects of the change in treatment recommendations to AziR rates in those regions. The high AziR levels seen between 2013 and 2018 were led by ST-12302. In 2021, while the number of isolates with ST-12302 has decreased, an ST cluster closely related to and including ST-12302 is responsible for 34.1% (n=89/261) of AziR isolates. ST-19875, the most prevalent ST in 2021, accounted for 23.8% (n=62/261) of AziR isolates, however, 75.8% (n=232/306) of isolates with this ST had azithromycin MICs of 1 mg/L, just one dilution beneath the resistance breakpoint MIC of 2 mg/L ((15)). The remaining AziR isolates are dispersed amongst various primarily unrelated STs with one to eight isolates in each.

As in 2020, the percentage of cultures with azithromycin MICs at or above the break point of 2 mg/L in 2021 was lower than in 2019. However, the proportion of *N. gonorrhoeae* cultures with a MIC of at least 1 mg/L increased significantly (*p*<0.001) in 2021 (Figure 5). Both ST-19875 and ST-17972, two of the most prevalent STs of 2021, have high proportions of isolates with an azithromycin MIC of 1 mg/L (75.8% and 58.3%, respectively). Although the Clinical and Laboratory Standards Institute’s recommended breakpoint for azithromycin is 2 mg/L ((15)), some countries including Australia have set their breakpoint at 1 mg/L, which is also the epidemiological cut-off value from the European Committee on Antimicrobial Susceptibility Testing ((37,38)). The shift to an azithromycin MIC of at least 1 mg/L in Canada should be monitored, as azithromycin is part of the dual therapy recommended for the treatment of gonorrhea.

While DGI cases in Canada decreased between 2020 and 2021, the decline was not significant, but is still an important concern. With the decrease in diagnosis of gonorrhea since the COVID-19 pandemic began, potentially from the impact that the pandemic measures had on STI testing, a further increase in DGI cases may be seen in the future and should be monitored.

## Limitations

Isolates and associated data submitted to NML by the provinces and territories are done on a voluntary basis and therefore not consistent across the country. This limits the overall interpretation of results as only a subset of isolates may have been submitted for testing from a region. Also, as the majority of gonococcal cases are diagnosed by NAATs, AMR rates may not be reflected accurately in this report and resistance rates may be under-reported.

Since the SAR-CoV-2 pandemic began in 2020, significantly fewer (*p*<0.001) *N. gonorrhoeae* cultures have been collected in Canada and made available to NML. Trends in incidence, AMR and molecular types may have been affected, especially for smaller provinces and territories with limited resources and capabilities.

## Conclusion

Gonorrhea remains an important public health concern due to its potential to cause infertility, pelvic inflammatory disease and DGI which can include dermatitis, arthritis, and in rare cases, endocarditis, meningitis or osteomyelitis ((13,14)). *Neisseria gonorrhoeae* has the ability to adapt to resist antimicrobials and this has been well documented ((3)).

Between 2017 and 2021, we have seen a number of trends in *N. gonorrhoeae* identified in Canada: 1) CxR isolates with a MIC equal to 1 mg/L; 2) a significant increase in the proportion of CeDS cultures; 3) AziR rates that exceed the WHO recommended levels required to change therapy and 4) a significant increase in the number of DGI cases across the country.

The continued surveillance of *N. gonorrhoeae* AMR trends is crucial to ensure national treatment guidelines are recommending the most effective therapies. Public health authorities can be informed of emerging AMR issues that could inform interventions when ongoing surveillance detects clonal outbreaks using molecular typing. The representativeness and interpretation of the current passive surveillance system data would be improved if epidemiological and laboratory data were linked. The Enhanced Surveillance of Antimicrobial-Resistant Gonorrhea, initiated in 2014, was developed to address these gaps ((39,40)).

## Supplemental material

These documents can be accessed on the Supplemental material file.
Table S1: Summary of the *Neisseria gonorrhoeae* culture and laboratory data received by the National Microbiology Laboratory, 2017–2021
Table S2: *Neisseria gonorrhoeae* antimicrobial resistance criteria
Table S3: Age of patient and isolation site of the *Neisseria gonorrhoeae* isolates tested at the National Microbiology Laboratory, 2021 (N=2,909)
Table S4: *Neisseria gonorrhoeae* cultures tested in each province, 2017–2021
Figure S1: Trends of multi-drug resistant *Neisseria gonorrhoeae* in Canada, 2017–2021
Figure S2: Trends of extensively drug-resistant *Neisseria gonorrhoeae* in Canada, 2017–2021
Table S5: All extensively resistant *Neisseria gonorrhoeae* isolated in Canada
Figure S3: Trends of prevalent *Neisseria gonorrhoeae* multi-antigen sequence types of isolates tested by the National Microbiology Laboratory, 2017–2021
Figure S4: Provincial distribution within *Neisseria gonorrhoeae* multi-antigen sequence types, 2021 (N=2,006)
Table S6: Ceftriaxone-resistant *Neisseria gonorrhoeae* identified in Canada, 2017–2021
